# Analysis of temporal evolution of quantum dot surface chemistry by surface-enhanced Raman scattering

**DOI:** 10.1038/srep29508

**Published:** 2016-07-08

**Authors:** İlker Doğan, Ryan Gresback, Tomohiro Nozaki, Mauritius C. M. van de Sanden

**Affiliations:** 1 Dutch Institute for Fundamental Energy Research (DIFFER), P. O. Box 6336, 5600 HH Eindhoven, The Netherlands; 2 Department of Applied Physics, Eindhoven University of Technology, P.O. Box 513, 5600 MB Eindhoven, The Netherlands; 3 Department of Mechanical Sciences and Engineering, Tokyo Institute of Technology, 2-12-1, O-Okayama, Meguro, 1528550 Tokyo, Japan

## Abstract

Temporal evolution of surface chemistry during oxidation of silicon quantum dot (Si-QD) surfaces were probed using surface-enhanced Raman scattering (SERS). A monolayer of hydrogen and chlorine terminated plasma-synthesized Si-QDs were spin-coated on silver oxide thin films. A clearly enhanced signal of surface modes, including Si-Cl_x_ and Si-H_x_ modes were observed from as-synthesized Si-QDs as a result of the plasmonic enhancement of the Raman signal at Si-QD/silver oxide interface. Upon oxidation, a gradual decrease of Si-Cl_x_ and Si-H_x_ modes, and an emergence of Si-O_x_ and Si-O-H_x_ modes have been observed. In addition, first, second and third transverse optical modes of Si-QDs were also observed in the SERS spectra, revealing information on the crystalline morphology of Si-QDs. An absence of any of the abovementioned spectral features, but only the first transverse optical mode of Si-QDs from thick Si-QD films validated that the spectral features observed from Si-QDs on silver oxide thin films are originated from the SERS effect. These results indicate that real-time SERS is a powerful diagnostic tool and a novel approach to probe the dynamic surface/interface chemistry of quantum dots, especially when they involve in oxidative, catalytic, and electrochemical surface/interface reactions.

Raman spectroscopy has been extensively used in single molecule detection with very high sensitivity thanks to an effect called surface-enhanced Raman scattering (SERS). SERS effect is based on localized surface plasmon resonances (LSPRs), which is observed when the incoming light exclusively interacts in resonance with dipolar surface plasmons of materials having free electrons^1^. These materials include thin films or nanostructures of Au^2^, Ag^3^,^4^, and Cu^5^, their oxides, and also semiconductors^6^. In SERS, resonance of optical fields and dipolar surface plasmon modes enable electromagnetically enhanced strong Raman scattering signals of adsorbed molecules in the surroundings of these enhancing materials^7^. The enhancement is a result of an increase in the Raman scattering cross section, which quantifies the probability of a scattering event to occur when the incident electromagnetic wave strikes on a molecule, and thus it is a measure of how high the Raman scattering intensity will be with respect to the incident electromagnetic wave. Together with other resonant processes (i.e., chemical enhancement), this brings the effective Raman cross-section (10^−30^ cm^2^/molecule) to a level of fluorescence cross-section (10^−16^–10^−15^ cm^2^/molecule) with extreme enhancements factors of 10^14^–10^15^ times^8^ (with the dominant enhancement of ~10^11^ from electromagnetic processes^1^), and enables the detection of Raman signal from single molecules^4^. Over the years, it has been shown that SERS is superior to other single molecule detection techniques like laser-induced florescence and low temperature optical absorption, because SERS effect provides highly resolved vibrational information and it is not affected from photobleaching^8^.

The ability of detecting single, or very low concentration of molecules have singled the SERS effect out as a particularly appealing technique to the researchers from the fields of biophysics/biochemistry^9^, bioanalytics^10^, chemical-sensing, and spectro (electro) chemistry^11^. Majority of the research focused on the detection of structural and chemical variation of small molecules using the SERS effect. In other words, the common use of SERS is that to approach or adsorb a molecule on a SERS-active nanostructured, or roughened surface, and detect the Raman-shifted enhancement signal from the adsorbate. On top of this, the extreme surface sensitivity of SERS could not only be used for detection of single molecules, but also be used as a surface/interface diagnostic method to analyze the chemical state of nanomaterial surfaces/interfaces. While plasmonic surfaces have been used to detect nanoparticle phonon modes^12^,^13^, there is no prior report that have used SERS effect to examine the quantum dot surface chemistry explicitly. Although the feasibility of employing a SERS-active substrate to monitor the chemical state of other nanomaterials surfaces/interfaces has not been exploited, the ability of extreme surface sensitivity offers a great potential on establishing SERS as a surface/interface chemistry analysis technique. Realization of such a surface analysis method with extreme sensitivity will obviously have a significant impact to nanotechnology-driven research due to the critically important surface properties, and surface-chemical dynamics of nanomaterials.

One of the most investigated nanomaterial systems that could benefit from the SERS based surface/interface analysis routes are silicon nanoparticles. Silicon nanoparticles, especially the nanoparticles in the quantum size regime, or silicon quantum dots (Si-QDs) have the potential to be critical components in future technological applications by virtue of their size dependent optical, catalytic, and electronic properties. Some of the featured applications of Si-QDs are light emitting diodes^14^,^15^, batteries^16^,^17^, CO_2_-free fuel production via water splitting^18^, bio-marking^19^, and solar cells^20^,^21^. Regardless of the nature of the application, as expected, surface chemistry plays a critical role on the efficiency, reliability, doping^22^, and compatibility of Si-QDs – as a result of increased surface-to-volume ratio and increased surface reactivity with respect to bulk Si. Having the ability to probe Si-QD surfaces and their surface chemical state is therefore of paramount importance to understand and optimize their functionalities in the applications they are being used. For instance, luminescence from surface functionalized Si-QDs^23^,^24^,^25^, electrochemical interaction of Si-QD surfaces with the electrolyte materials in batteries^26^, reactivity of Si-QD surfaces in water during water splitting^18^, or their biocompatibility^27^ can be better understood and controlled using an *in situ* temporal diagnostic method with molecular level surface chemistry sensitivity.

Harnessing the SERS effect could open a whole new research area on the surface chemistry analysis of Si-QDs. This work deals with using the SERS effect to monitor the chemistry of Si-QD surfaces/interfaces when they are located on a SERS-active surface. For the SERS analyses, we employed free-standing, unoxidized Si-QDs with chlorine and hydrogen terminated surfaces. We observed a clearly enhanced Raman scattering signal from these Si-QDs spin-coated on silver oxide, i.e., Ag/Ag_2_O (Ag_2_O is the natural oxide of silver when it is oxidized by molecular oxygen^28^,^29^), SERS-active surfaces in an oxygen-free environment. The SERS signal revealed information from the surface termination of as-synthesized Si-QDs, involving Si-Cl_x_ and Si-H_x_ modes. We also observed that, upon oxidation –via exposing Si-QDs to ambient conditions– monitoring the temporal evolution of Si-QD surface chemistry is possible. In addition, SERS spectra revealed the crystalline morphology of Si-QDs from the transverse optical (TO) phonon mode of crystalline silicon. These findings imply that, real-time SERS is a powerful diagnostic tool to probe the surface chemistry of Si-QDs during their interaction with the surrounding medium.

## Results

### SERS Substrates

**Figure 1:**
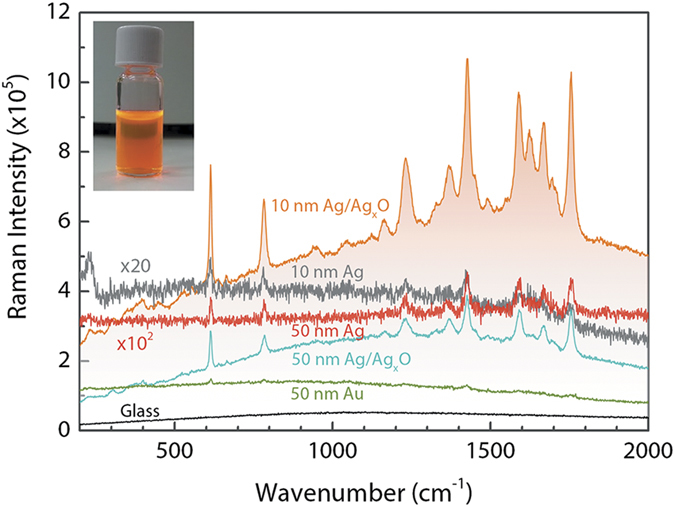
Comparison of SERS signals of 10^−7^ M R6G from bare glass and from various thin films with different thicknesses. The highest enhancement was obtained from Ag/Ag_2_O thin films with 10 nm thickness. The broad background enhancement is due to the fluorescent emission upon decomposition of Ag_2_O films under laser light exposure. Picture in the graph shows R6G in a glass vial.

An important consideration on obtaining an efficient SERS effect is the right matching of the excitation wavelength with the plasmonic resonance window of the SERS material to be used. In addition, the type and morphology of the material, and the feasibility of production are the other important considerations. The classical SERS materials, Cu, Au and Ag are the mostly used ones with different shapes^7^,^30^ and morphologies such as spherically shaped nanoparticles^31^,^32^, nanowires^33^,^34^, roughened and oxidized thin films^35^,^36^,^37^,^38^. Here, we deposited thin films of Ag on glass substrates using thermal evaporation technique, which was located in a nitrogen purified glovebox – so that the thin films were processed in an air-free environment. It is known that Ag has a plasmonic resonance window in the range 400–1000 nm^11^, where it supports SERS. Using 514 nm laser light as the excitation wavelength, we expect to have a clear, characteristic enhancement of the Raman signal from Ag thin films. Figure 1 demonstrates the SERS spectrum from 10^−7^ M R6G in ethanol, spin-coated on Ag thin films. For comparison, R6G on bare glass substrates and on Au thin films are also demonstrated. In contrast to the R6G signal from bare glass substrates, which does not display any spectral feature, a clear SERS signal of R6G was observed both from Ag and Au at wavenumbers 614.2, 782.9, 1426.7 and 1753.0 cm^−1^. However, as expected, the SERS enhancement is much more evident from Ag thin films with respect to Au thin films under 514 nm light, due to fact that the 514 nm excitation falls within the plasmon resonance range of Ag, rather than Au (Au has a plasmon resonance in the range 600–1250 nm^11^). Observed main R6G vibrational modes are: aromatic C-C stretching modes in the range 1350–1750 cm^−1^, C-C bending mode around 1200 cm^−1^, and C-H bending mode around 1150 cm^−1^ 33,39. Even higher SERS signals with additional modes of R6G were observed when the Ag thin films were kept in ambient conditions for 2 minutes for oxidation before spin-coating it with R6G in the glovebox. Under ambient conditions, Ag thin film was oxidized, ended up with a shiny white appearance. In this case, weak C-C stretching modes were also observed from R6G at 1140, 1450 and at 1680 cm^−1^ 33,40, which proves the improved enhancement of Ag/Ag_2_O thin films with respect to Ag thin films. As a function of film thickness, we found that the highest enhancement was observed from 10 nm Ag/Ag_2_O films (the enhancement observed from 100 nm films were also similar to that of 50 nm films, therefore it is not shown here for brevity). Together with the Raman enhancement, we also observed a broad fluorescence signal from R6G spin-coated on Ag/Ag_2_O thin films^1^,^41^.

Here, we should justify the role of Ag film thickness, and oxidation of Ag film on the optimization of SERS effect. In order to achieve SERS effect from structures that are responsible for the plasmonic enhancement, their feature sizes should be considerably small with respect to the excitation wavelength. In general, in order to achieve a SERS activity, the feature size should lie in the range of a few nanometers to hundreds of nanometers^1^,^42^. If features responsible for plasmonic enhancements are of the order of the excitation wavelength or larger, dipolar plasmons are not exclusively excited anymore. Instead of this, higher order plasmon modes are excited, which have non-radiative characteristics, unlike the radiative dipole modes. This results in a complete extinction of the SERS signal. In the literature, a number of studies have been reported on the effect of silver film thickness on SERS intensity. These studies report the observation of SERS effect from a wide range of film thicknesses starting at 2 nm up to 300 nm^37^,^38^,^43^,^44^,^45^. According to one of these reports, the maximum enhancement was observed from Ag when the features were around 50 nm^44^. This is also the case for Ag thin films shown in Fig. 1 (i.e., 50 nm Ag thin film has a better-defined R6G signal with respect to 10 nm Ag film). However, when the thin films are oxidized, the maximum R6G intensity is observed from 10 nm Ag/Ag_2_O films, which is almost a factor of three higher with respect to 50 nm Ag/Ag_2_O thin films. Similarly, others have also reported high SERS enhancements from silver oxide thin films in the thickness range 5–15 nm rather than thicker films^37^,^38^.

**Figure 2:**
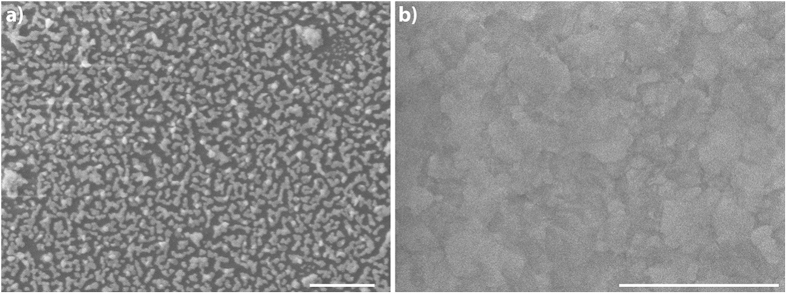
SEM images of Ag/Ag_2_O thin films. (**a**) View of 10 nm Ag/Ag_2_O film. Island-like morphology is clearly seen. Dark color corresponds to glass substrate and lighter color corresponds to the islands. (**b**) View of 50 nm Ag/Ag_2_O film reveals continuous and rough morphology. Scale bars are 1 μm.

The increased SERS effect upon oxidation of Ag thin films is related to the surface roughness modification and decomposition of Ag_2_O layer under laser irradiation. Oxidizing the Ag surface induces additional roughness, which further leverages the localized surface plasmon resonance (LSPR) intensities. Decomposition of Ag_2_O and formation of Ag clusters with Ag^+^ ions upon laser light exposure is known as one of the main reasons of SERS effect observed from these films^37^,^38^. The created Ag^+^ ions act as a “hot” spot and form a charge-transfer complex with the target molecule adsorbed or nearby this spot, which gives rise to R6G SERS spectra on Ag_2_O layers. Observation of higher SERS enhancement from 10 nm films with respect to 50 nm films is a result of more efficient decomposition and Ag cluster formation in thinner films. Figure 2 demonstrates that 10 nm Ag/Ag_2_O film is in the form of islands, wile 50 nm Ag/Ag_2_O film has a rather continuous and rough morphology. Decomposition of 10 nm Ag/Ag_2_O could promote isolated “hot” spots, which results in higher enhancement factors. It is also known that silver oxide gives rise to a strong fluorescence at the background of SERS spectra of target molecules^37^,^46^. In Fig. 1, the broad background features only appear in oxidized films, which is an evidence of charge-transfer complexes formation. In this respect, observation of more intense luminescence from 10 nm Ag/Ag_2_O thin films is also an additional evidence of more efficient Ag_2_O layer decomposition, resulting in higher enhancements.

The oxidation of Ag by molecular oxygen is governed by Fick’s law of diffusion. At room temperature and under atmospheric conditions, growth of natural Ag_2_O layer on Ag in a pure oxygen at room temperature goes up to 10–20 Å during the first hour, and stays unchanged further in time^28^. Considering the oxidation time of 2 minutes, the oxide layer thickness is well below 10 Å. In conclusion, from the preparation of the SERS substrates, we obtained the highest R6G SERS enhancement from 10 nm Ag/Ag_2_O thin films. Using Ag/Ag_2_O films is therefore proved as a feasible means of performing SERS experiments on temporal evolution of Si-QD surfaces during oxidation.

### FTIR analysis of Si-QDs

**Figure 3:**
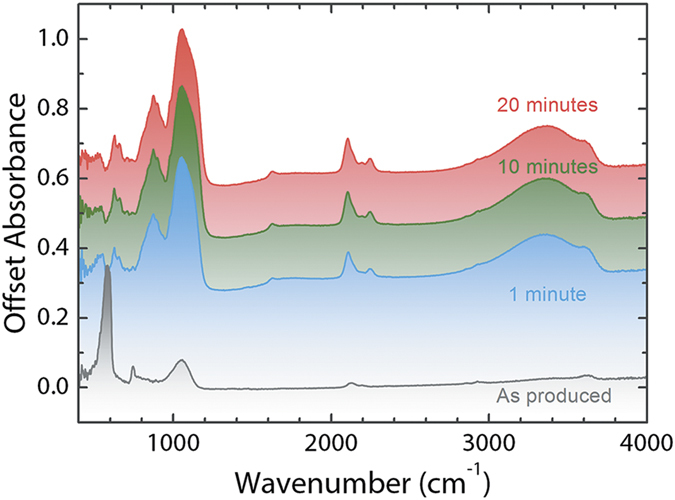
FTIR spectra of Si-QDs synthesized in a very high frequency non-thermal plasma. The spectra shows the aging of as-synthesized Si-QDs up to 20 minutes.

Fourier transform infrared spectroscopy (FTIR) was used to probe the chemistry of synthesized Si-QDs. Figure 3 demonstrates the as-synthesized Si-QDs and the temporal evolution of their chemistries under ambient conditions. In the FTIR spectra we clearly observed the surface modes related to hydrogen, chlorine, and oxygen. Hydrogen related modes were observed in the regions 2000–2200 cm^−1^ and 800–950 cm^−1^. These modes are ascribed to Si-H_x_ stretching modes and bending modes, respectively. Interestingly, we observed an increase of the Si-H_x_ modes intensity upon exposure to air, which was also observed previously during the oxidation of chlorinated Si-QDs^47^,^48^. The reason of this increase could be due to the formation of highly strained Si-OH form from water, and its subsequent reconstruction to Si-O-Si-H form. Chlorine related modes were observed with highest intensity from the as-synthesized Si-QDs and their intensities decreased with exposure to air. These modes of Si-Cl_x_ are located at 575 cm^−1^ region^47^. No OH groups were probed from the surfaces of as-synthesized Si-QDs. As expected, the peak intensity of OH groups at 3300 cm^−1^ increased with longer exposure times to air. The observed Si-O-Si modes before exposing Si-QDs to air, from the range 1000–1150 cm^−1^, could be due to residual moisture contamination in the glovebox, or etching of the quartz reactor walls during the quantum dot synthesis^47^. The intensity of Si-O-Si mode increased with prolonged exposure to air.

### SERS of Si-QDs

**Figure 4:**
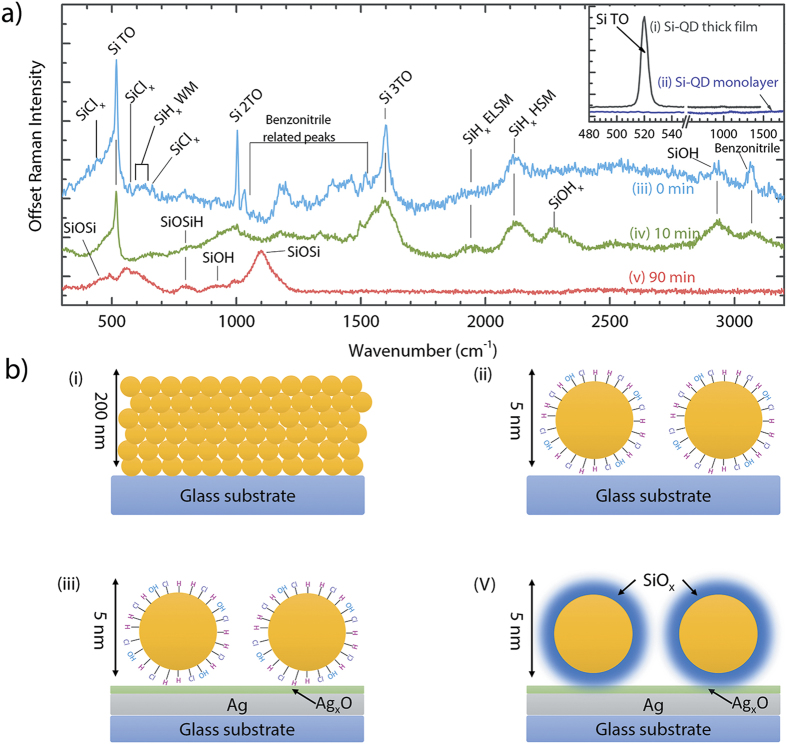
SERS analyses of Si-QDs on Ag/Ag_2_O thin films as a function of air-exposure time: 0 min (non-oxidized, air free analysis 4**a**-iii and 4**b**-iii), 10 min (4**a**-iv), and 90 min (4**a**-v, and 4**b**-v). Inset shows a measurement from Si-QDs on glass substrates in the form of a thick film (4**a**-i, and 4**b**-i), and with the same quantum dot density as SERS analyses (4**a**-ii, and 4**b**-ii).

Raman spectra of Si-QDs spin-coated on Ag/Ag_2_O SERS substrates are demonstrated in Fig. 4. First, we stress that Si-QDs deposited as a thick film (thickness of 200 nm as in the FTIR measurements) on bare glass substrates (Fig. 4a-i,b-i) have a clear Raman signal at 519.8 cm^−1^ with an estimated size of 5.3 nm according to the phonon confinement model^49^. For the thick Si-QD film, the rest of the spectrum does not have any additional features as seen in the inset of Fig. 4a-i. On the other hand, Si-QDs, with the same number density as used in the SERS measurements, measured on bare glass demonstrated a flat character in a broad spectral range, concluding that the scattering intensities from Si-QDs are beyond the detection limit of the Raman spectrometer before any enhancement (Fig. 4a-ii,b-ii). These two measurements demonstrate that a monolayer of Si-QDs is not detectable with standard Raman spectroscopy, and only when the Si-QD layer is thick enough, a single peak corresponding to crystalline Si (*c*-Si) is observed. Furthermore, no information related to the Si-QD surface chemistry can be obtained from the standard Raman analyses.

Si-QDs analyzed on Ag/Ag_2_O substrates show a distinct Raman spectrum, clearly showing the effect of SERS from Si-QD surfaces. We discuss the detected peaks as a function of air exposure time, namely from 0 minute (non-oxidized, air-free) to 90 minutes. Unlike the Raman spectrum of Si-QDs on glass regions of the substrate, a monolayer of non-oxidized Si-QDs on Ag/Ag_2_O regions (0 min) has a very clear footprint of Si-QD TO phonon mode at 519.8 cm^−1^ proving the enhancement as seen in Fig. 4a-iii. In addition, second (2TO) and third (3TO) order transverse optical modes were also detected at ~1000 cm^−1^ and at 1600 cm^−1^, respectively, which was not observed from Si-QD thick film (Fig. 4a-i). This observation further demonstrates the enhanced electron-phonon coupling as a result of SERS effect^50^. Apart from the TO modes, the rest of the spectrum is rich of surface-related modes: additional features observed from the surface of Si-QDs were Si-Cl_x_ modes around 450, 540, and 650 cm^−1^ 51,52, Si-H_x_ wagging modes in the region 600–630 cm^−1^ 51, and Si-H_x_ stretching modes in the region 1900–2200 cm^−1^ 51, which clearly shows the surface termination of Si-QDs by chlorine and hydrogen. A striking observation is the ability of SERS resolving Si-H_x_ stretching modes (1900 cm^−1^ is the extreme low stretching mode (ELSM), and 2100 cm^−1^ is the high stretching mode (HSM)), which cannot be distinguished in a single FTIR measurement as the signal comes both from the surface and from the sub-surface of Si-QDs^53^,^54^. This observation indicates that only ELSM and HSM of Si-H_x_ were present on the quantum dot surfaces (as the sub-surface signal is greatly reduced or even ruled out since the SERS intensity scales with *d*^−10^ with distance from the LSPR region, which makes it unlikely to detect Si-H modes inside of Si-QDs^55^). Additional peaks in the region 1200–1600 cm^−1^ and 3050 cm^−1^ indicated the presence of benzonitrile fragments attached to the surface of Si-QDs. Particular location of benzonitrile modes are at 1270, 1320, 1390, 1465, 1530, and 1570 cm^−1^. The peak at 3050 cm^−1^ indicates the bonding of hydrogen to the CN group of benzonitrile^56^. An Si-OH presence was also detected at 2920 cm^−1^, which is possibly due to the residual water adsorbed on Si-QD surfaces during sample preparation in nitrogen purified glovebox.

After 10 minutes of oxidation of Si-QDs on SERS substrates (Fig. 4a-iv), a different SERS spectrum was observed with respect to the non-oxidized quantum dot surfaces. Si-QD TO modes were observed with less intensity, and oxidation-related peaks become visible: Si-O-Si-H mode at 800 cm^−1^, Si-O-H_x_ mode at 2250 cm^−1^, and an increased Si-OH signal at 2920 cm^−1^. As a result of oxidation, Si-Cl_x_ and benzonitrile modes disappeared. In addition, Si-H_x_ modes in the range 1900–2100 cm^−1^ were detected at higher intensities upon oxidation, similar to the FTIR analyses shown in Fig. 3, and to the previous reports on oxidation of chlorine terminated Si-QD surfaces^47^,^48^. After 90 minutes of oxidation (Fig. 4a-v,b-v), most of the vibrational modes that belong to Si, and the broad feature located in the region 1700–3000 cm^−1^ have disappeared. On the other hand, additional oxidation-related modes were detected in the range 450–1200 cm^−1^, similar to the FTIR spectra shown in Fig. 3. These are Si-O-Si modes at 450 and 1100 cm^−1^, Si-O-Si-H mode at 800 cm^−1^ (with an increased intensity with respect to 10 minutes of oxidation), and Si-OH stretching mode at 900 cm^−1^.

**Figure 5:**
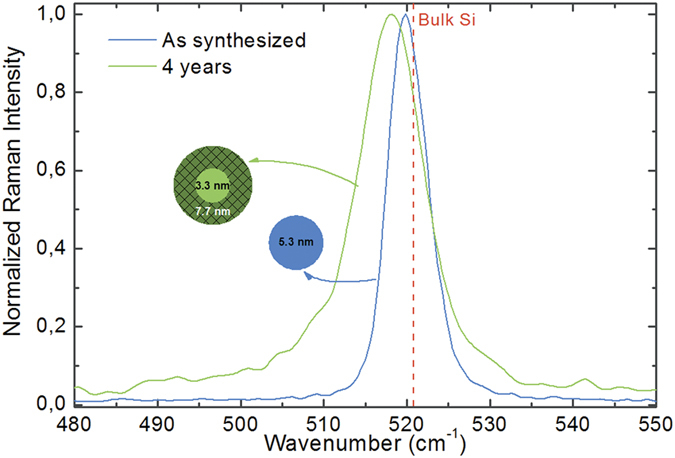
Raman spectra of as-synthesized Si-QDs (same as Fig. 4a–i) and Si-QDs remained under ambient conditions for four years. Oxidation is accompanied with a reduction of the crystalline core size, which appears as a shift to lower wavenumbers in the Raman spectrum. Estimated oxide thickness as about 2.2 nm.

In addition to the formation of charge-exchange complexes between Ag/Ag_2_O and Si-QD surfaces. Another mechanism that plays an additional role is the excitation of gap modes between the Si-QD and Ag/Ag_2_O thin film, creating a hot spot of concentrated electromagnetic energy. This hot spot acts as an antenna, where the surface plasmon polaritons propagate from metal to Si-QD surface. The Raman intensity of the molecules (surface molecules of Si-QDs) residing at this hot spot is therefore greatly enhanced^57^. The reason of disappearance of the main features observed in the SERS spectra in Fig. 4a-iii after oxidation of Si-QDs (Fig. 4a-v) is due to the increased oxide thickness of Si-QDs, which increases the distance between the LSPR zone and the Si-QD surface within the oxide shell, decoupling the plasmonic enhancement and surface plasmon polariton propagation. According to our previous report, the approximate oxide thickness grown on Si-QDs after 90 minutes of exposure to air is about 1.5 nm for the originally chlorine terminated Si-QDs, which is in line with the Cabrera-Mott mechanism for oxide growth on quantum dot surfaces (During the oxidation experiment, Si is the main source of oxide growth as the oxide thickness on Ag does not increase considerably within the course of 90 minutes)^47^,^58^. The enhancement exhibits an ***E***^4^ dependency (***E*** is the electric field), which leads to a distance dependency of *d*^−10^ for the intensity of SERS effect (*d* is the distance between the hot spot and Si-QD surface). Considering this strong distance dependence of SERS intensity, the distance between the LSPR zones and Si-QD surfaces in the oxide shell kills the SERS effect, ending up with a SERS signal only from the top oxide surface of Si-QDs as observed after 90 minutes. It is important to note that the disappearance of the crystalline Si-TO Raman mode is due to the oxide layer growth on Si-QD, and not a complete oxidation of Si-QDs into silicon oxide nanoparticles. To support the existence of crystalline Si-QD core, Fig. 5 demonstrates the comparison of as-synthesized Si-QDs, and Si-QDs that were exposed to the ambient conditions for more than four years. As synthesized Si-QDs are the same as demonstrated in Fig. 4a-i, with a TO Raman mode of 519.8 cm^−1^ and an average size of 5.3 nm according to the phonon confinement model^49^. Same Si-QDs still demonstrate a clear crystalline Si TO Raman mode after four years, with a shifted peak shape. The shift is related to the relative decrease in the crystalline core size, which is expected as a result of oxidation. The observed shift is 517.8 cm^−1^, which corresponds to a size of 3.3 nm^49^. Considering that during oxidation, about 50% of the oxide layer thickness is consumed from Si-QD surface, the estimated overall oxide layer thickness was found as 2.2 nm.

We have seen that in the case of standard Raman scattering, as demonstrated in Fig. 4a-i, the field-induced dipole moment in Si-QDs allow contributions from the transverse optical mode of crystalline silicon. However, abovementioned results demonstrate that a number of additional Raman active modes appeared at SERS spectra. The origin of these additional modes is a result of relaxation of the selection rules for Raman active modes as a result of the plasmonic enhancement in the vicinity of the Ag/Ag_2_O film. The dominant contribution at standard Raman scattering originates from the first order electric-dipole term in the dipole moment, and the contributions from the second order electric-quadrupole and the third order magnetic-dipole terms are Raman silent as their intensities are negligible with respect to the intensity of the Raman active modes. However, in the vicinity of a nanostructured plasmonic surface, the intensities of these higher order modes become comparable to the intensity of the first order related modes, making them Raman active as well. Therefore, the resurrection of these new Raman active modes is a result of relaxed selection rules due to the inhomogeneous enhancement at the close proximity of the plasmonic surface^42^,^59^,^60^. This is also the reason why these modes carry extremely surface-sensitive information, i.e., they only extend a very short range away from the plasmonic surface.

Using the SERS effect to probe the surface chemistry of Si-QDs can potentially be used in the research, where Si-QD surface chemistry plays a critical role. A topic featuring the critical role of Si-QD surfaces is the research of Si-QD based lithium-ion batteries (LIBs), where Si-QD surfaces involve in electrochemical interactions with the electrolyte material during battery operation. Si-QDs are considered as one of the potential next generation anode materials due to their ten-folds charge storage capacity (~4000 mAh/g) when compared to the commercial carbon-based anodes (375 mAh/g) in today’s LIBs. The challenge on realizing Si-QD based LIBs is controlling the volumetric expansion of Si-QDs upon lithiation, and continual solid electrolyte interface (SEI) formation after each charge/discharge cycle as a result of decomposition of electrolyte material on Si-QD surfaces. SEI is known to trap Li and consume Si in an irreversible way, hampering the useable capacity of Si. Currently, promoting an in-depth understanding of SEI formation on Si-QD surfaces is lacking, due to the limited availability of the real-time *in situ* diagnostic tools with extreme surface sensitivity. We propose that, in this point, SERS can be used as a real-time diagnostics for monitoring the electrochemical reactions between Si-QD surfaces and electrolyte. The possibility of employing a nanostructured metal substrate as current-collector for the anode and depositing a monolayer of Si-QD/electrolyte mixture can enable real-time SERS analyses when a Si-QD-based LIB is in operation. Establishing such a diagnostics with extreme surface sensitivity can potentially reveal the underlying mechanism leading to the formation of SEI on Si-QD surfaces and suggest strategies to prevent its formation.

## Conclusion

In summary, we have demonstrated the SERS effect from Ag/Ag_2_O films and used this effect to monitor the temporal evolution of surface chemistry of Si-QDs during oxidation. Real time monitoring of quantum dot surfaces exposed to air revealed the increased oxygen incorporation on quantum dot surfaces, and the decreased silicon-hydrogen and silicon-chlorine modes, which covered the surface of the as-synthesized quantum dot surfaces. After 90 minutes of air exposure, the oxide layer on Si-QDs were about 1 nm, which resulted in a spectrum solely containing oxidation related modes. Vanishing of the previously observed features from the non-oxidized Si-QDs was related with the oxide thickness, which resulted in decoupling of the LSPR zones and the quantum dot surfaces beneath the oxide layer. These observations suggest that SERS can effectively be used for chemical analysis of Si-QD surfaces, which is of critical importance for achieving the desired stability and functionality of Si-QDs for the future technological applications.

## Methods

### Synthesis and Solution Processing of Si-QDs

**Figure 6:**
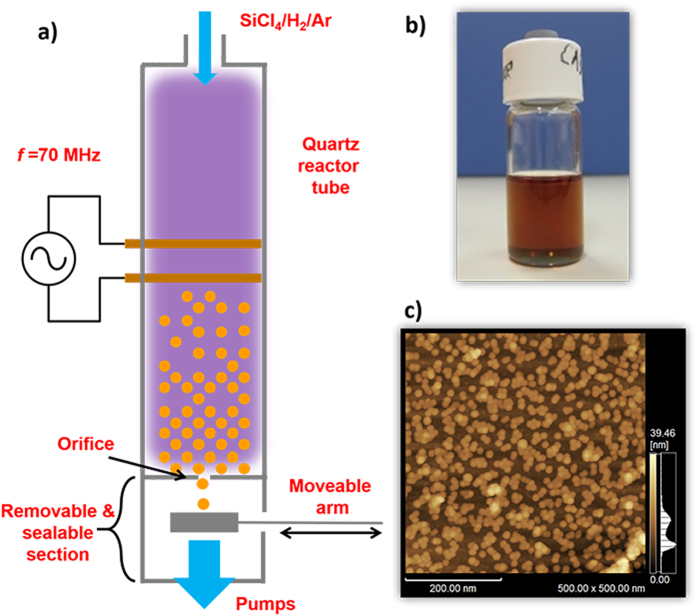
Preparation and characterization of Si-QDs. (**a**) A very high frequency (70 MHz) non-thermal plasma is generated using two copper electrodes surrounding a quartz tube, which is employed as a reactor. (**b**) Si-QDs dispersed in benzonitrile with a density of 1 mg/ml. (**c**) AFM image of Si-QDs after spin-coating. For this image, a Si-wafer was used, and coated using the same method as the preparation of SERS substrates.

Free standing Si-QDs were synthesized in a very high frequency (VHF, 70 MHz) non-thermal plasma using a SiCl_4_/H_2_/Ar gas mixture as described elsewhere^47^. Formation of Si-QDs are favored by dissociation of SiCl_4_ molecules through electron impact followed by nucleation and subsequent growth. During the synthesis, the mean size of Si-QDs is controlled by tuning the residence time of the gas flow in the plasma, which resulted in diameters of about 5.3 nm. During the deposition, Si-QDs were collected onto a moveable mesh as illustrated in Fig. 6(a). The sealable and removable quantum dot collection section of the reactor was decoupled keeping the inside volume in the pressurized Ar atmosphere, and transported into a nitrogen purified glovebox (<1 ppm oxygen and water) for air-free processing. Here, Si-QDs were exposed to ultrasonic treatment while they are dispersed in anhydrous benzonitrile (Sigma-Aldrich) to form a stable, colloidal quantum dot ink. Prepared colloidal ink is diluted to a quantum dot density of 1 mg/ml (Fig. 6(b)). From this solution, 0.1 ml is drop-casted and spin-coated on SERS substrates, and on glass and silicon substrates (for control experiments) with dimensions of 1 × 2 cm, which resulted in a monolayer of Si-QDs with similar surface coverages. Figure 6(c) is an atomic force microscope (AFM) image that demonstrates a monolayer of Si-QDs spin-coated on a silicon substrate. Spherical Si-QDs are clearly visible on the dark brown-colored surface of silicon substrate. General view of the AFM image demonstrates small groups of isolated Si-QDs on the surface, apart from some minor groups composed of Si-QDs laying on top of each other (right bottom part, group of Si-QDs with lighter color).

### Preparation of the SERS substrates

**Figure 7:**
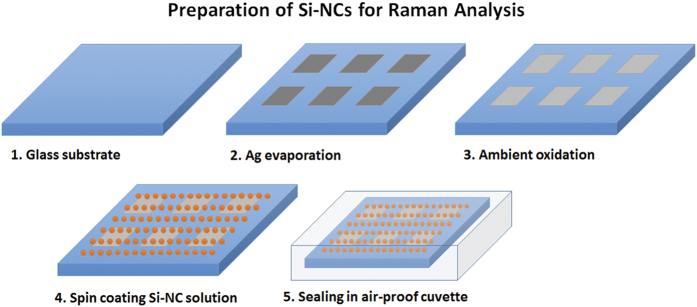
Preparation of SERS substrates and spin coating of Si-QD colloidal inks. Step 3 is exclusively for Ag_2_O formation. For Ag thin films, this step is skipped.

Before employing SERS substrates for the analysis of Si-QDs, we performed a series of optimization studies of these substrates to demonstrate that Ag/Ag_2_O thin films have the highest enhancement for our experimental conditions. For this aim, rhodamine 6 G (R6G), with a concentration of 10^−7^ M in ethanol solution, was used. R6G is known for its extremely high response to SERS effect, and that the intensity of R6G Raman spectra will indicate the best SERS substrate to be used for Si-QD analysis. R6G was spin-coated on Ag and Ag/Ag_2_O thin films, Au thin films (for comparison of plasmonic enhancement with respect to Ag and Ag/Ag_2_O at the 514 nm excitation of the Raman laser used in this study), and on bare glass substrates (for control experiments). Figure 7 shows the preparation sequence of the SERS substrates. In a nitrogen purified glovebox, Ag thin films were deposited through a square shaped mask on glass substrates in a thermal evaporator. Film thicknesses were monitored by a thickness monitor and were estimated as 10, 50 and 100 nm. Later, Ag coated glass substrates were kept in ambient conditions for 2 minutes to allow formation of a top Ag_2_O layer. Following oxidation, Ag/Ag_2_O samples were processed in the glovebox. Si-QD colloidal ink was drop-casted on Ag/Ag_2_O SERS substrates (as the highest enhancement of R6G was observed from these thin films, which will be discussed below) and later on spin-coated until all the benzonitrile is dried. Finally, R6G and Si-QD spin-coated SERS substrates were put in sealable UV-grade quartz cuvettes for Raman spectroscopy measurements.

### Post analysis techniques

Fourier transform infrared spectroscopy (FTIR, JASCO 6100) analyses were conducted by using Si-QDs, which were directly deposited from the plasma onto a crystalline silicon substrate with enough thickness (200 nm) to collect sufficient signal. The mesh grid with Si-QDs were put inside a vacuum chamber with thallium bromoiodide (KRS-5) window to allow performing measurements in the transmission mode. For Raman spectroscopy measurements, SERS substrates with R6G, and with Si-QDs were used respectively, as described above. The Raman laser had a wavelength of 514 nm. The gratings used for the measurements had 1200 lines/mm. Scanning electron microscopy (SEM, JEOL) images of Ag/Ag_2_O thin films were acquired under an electron acceleration voltage of 5.0 kV.

## Additional Information

**How to cite this article**: Doğan, İ. *et al*. Analysis of temporal evolution of quantum dot surface chemistry by surface-enhanced Raman scattering. *Sci. Rep*. **6**, 29508; doi: 10.1038/srep29508 (2016).
